# 3D slicer combined with neuroendoscope in treatment of a distal segment aneurysm of the anterior choroidal artery complicated intraventricular hemorrhage: A case report and literature review

**DOI:** 10.1016/j.heliyon.2023.e16193

**Published:** 2023-05-18

**Authors:** Long Zhou, Yuting Ren, Zhiyang Li, Huikai Zhang, Hangyu Wei, Ping Song, Li Cheng, Wenju Wang, Lun Gao, Pan Lei, Qiuwei Hua, Qianxue Chen, Jinjian Zhou, Guoliang Li, Qiang Cai

**Affiliations:** a Department of Neurosurgery, Renmin Hospital of Wuhan University, Wuhan, Hubei 430223, China; b Department of Neurosurgery, Hubei Yunmeng County Hospital of Traditional Chinese Medicine, Yunmeng, Hubei 432500, China; c Department of Critical Care Medicine, Eastern Campus, Renmin Hospital of Wuhan University, Wuhan, Hubei 430223, China; d Department of Anesthesiology, Renmin Hospital of Wuhan University, Wuhan, Hubei 430223, China; e Department of Neurosurgery, People's Hospital of Dongxihu District, Wuhan, Hubei 430040, China

**Keywords:** 3D slicer, Transcranial neuroendoscope, Distal segment aneurysm of the anterior choroidal artery, Intraventricular hemorrhage

## Abstract

**Introduction:**

Pure ventricular hemorrhage is often secondary to Moyamoya disease, rarely caused by rupture of ventricular aneurysm. The surgical treatment of the latter is very challenging. 3D Slicer reconstruction technology can accurately locate small intracranial lesions and combined with minimally invasive surgery with transcranial neuroendoscope is a new attempt to treat the above diseases.

**Case presentation:**

We report a case of pure intraventricular hemorrhage secondary to rupture of a distal segment aneurysm of the anterior choroidal artery. Brain computed tomography (CT) before admission showed pure ventricular hemorrhage, and brain CT angiography (CTA) before operation showed a distal segment aneurysm of the anterior choroidal artery. We used 3D Slicer reconstruction and precise location of the focus before the operation and used the minimally invasive surgery technique with transcranial neuroendoscope to completely remove the hematoma in the ventricle, and found the responsible aneurysm located in the ventricle.

**Conclusion:**

Pure intraventricular hemorrhage requires vigilance against the distal segment aneurysm of the anterior choroidal artery. At present, conventional microscopic craniotomy and intravascular interventional therapy have limitations, and 3D Slicer reconstruction and precise positioning technology combined with transcranial neuroendoscope minimally invasive surgery may be a good choice.

## 1 Introduction

Intraventricular aneurysms are a rare subtype of intracranial aneurysms, often located in the lenticular artery or the anterior/posterior choroidal artery, and most of them are associated with moyamoya disease [1]. The incidence of anterior choroidal artery aneurysms is relatively low, accounting for about 2% to 5% of intracranial aneurysms [2]. They are usually located at the origin of the anterior choroidal artery, while aneurysms located at the distal segment of the anterior choroidal artery are extremely rare. At present, there are about 31 cases reported in the literature [3], and fewer aneurysms occur in the posterior choroidal artery, with only 11 cases reported in the literature [4]. In the past, conservative treatment was often used for such aneurysms, of which about 60% had a poor prognosis due to clinical progressive deterioration or rebleeding. With the development of micro-neurosurgery, surgical intervention for these kinds of aneurysms had been gradually attempted. The surgical methods include craniotomy microscope surgery, intravascular interventional therapy and neuroendoscopic surgery [3,5]. Our team recently completed the treatment about a case of pure intraventricular hemorrhage secondary to rupture of a distal segment aneurysm of the anterior choroidal artery using 3D slicer reconstruction and localization system combined with neuroendoscopy. The prognosis is good.

## 2 Clinical presentation

**Fig. 1:**
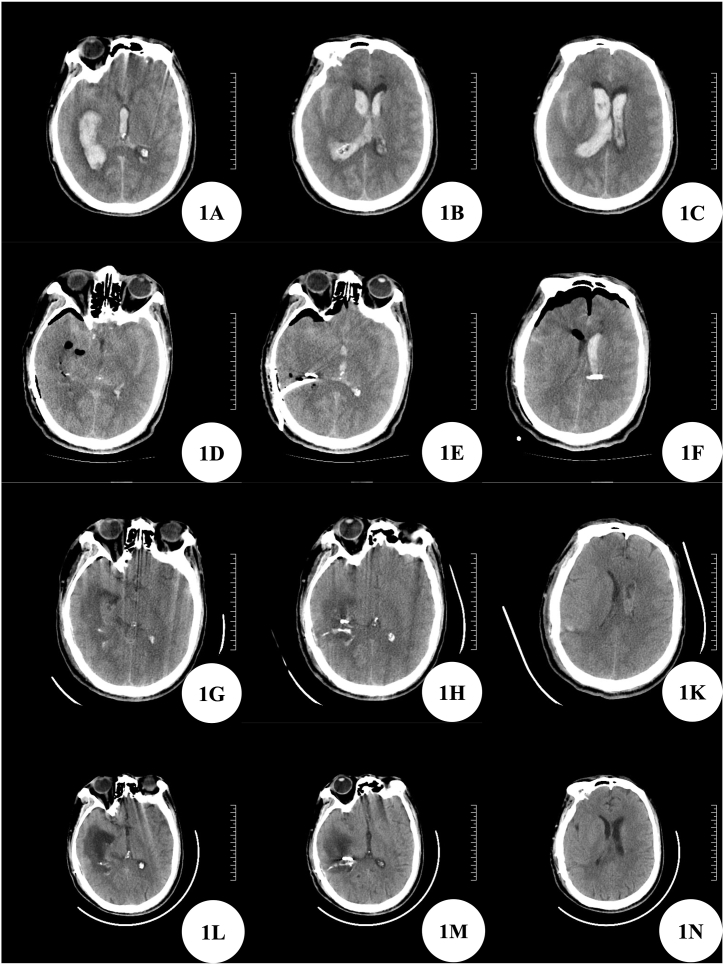
1A–C: Preoperative Brain CT; 1D–F: Brain CT on the day after operation; 1G–K: On the 8th day after operation, Brain CT was performed one day before lumbar cistern drainage; 1L–N: Brain CT before discharge.

**Fig. 2:**
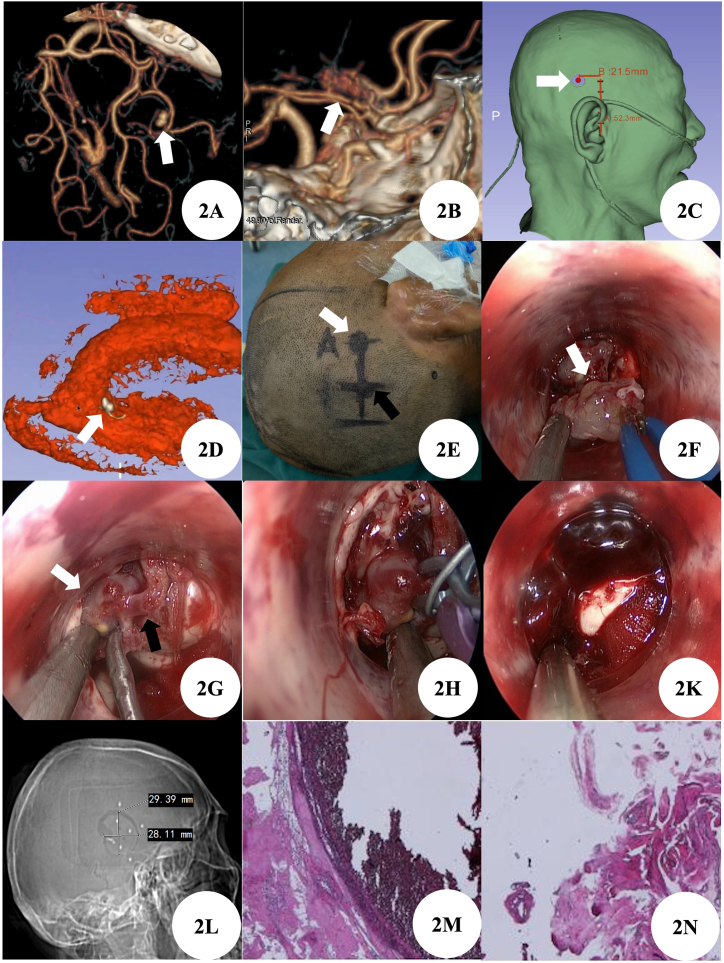
2A：Preoperative Brain CTA showed a distal segment aneurysm of the right anterior choroidal artery; 2B: Preoperative Brain CTA showed puff of smoke sign; 2C: 3D slicer reconstruction of the aneurysm surface projection; 2D: Relationship reconstructed by 3D slicer between the aneurysm and ventricular system; 2E: According to the projection of the body surface, locate the aneurysm (white arrow) and design the surgical incision line (black arrow); 2F: Choroid plexus papilloma was seen under neuroendoscope during operation; 2G: Aneurysm (white arrow) and Parent artery (black arrow) were seen under neuroendoscope during operation; 2H: Clipping aneurysm by using neuroendoscopic; 2K: Removaling intraventricular hematoma by using neuroendoscopic; 2L: Postoperative Brain CT showed that the size of bone window was about 3cm; 2 M: Pathological examination of postoperative aneurysm; 2 N: Pathological examination of choroid plexus papilloma after operation.

A 59-year-old male was admitted to our hospital for 7 hours due to sudden severe headache and disturbance of consciousness. When admitted, he was in a coma with a Glasgow Coma Score (GCS) of 5. The patient had no special medical history. Brain CT before admission indicates ventricular hemorrhage. After admission, our emergency reexamination of Brain CT showed intraventricular hemorrhage and subarachnoid hemorrhage (Fig. 1A–C). At the same time, the completion of head and neck CTA suggested that a distal segment aneurysm of the right anterior choroidal artery (Fig. 2A), the right middle cerebral artery and its branches were sparsely developed, and there were multiple capillary shadows around M1 segment, which was considered as puff of smoke sign (Fig. 2B). According to the 3D slicer reconstruction of the aneurysm, the lateral ventricle structure, and the relationship with the scalp (Fig. 2C–D), we designed a straight incision above the back of the right ear (Fig. 2E) and used the minimally invasive small incision surgery assisted by neuroendoscope to successfully clamp the distal segment aneurysm of the right anterior choroidal artery located in the lateral ventricle (Fig. 2G–H), while simultaneously removing the lateral ventricle hematoma (Fig. 2K). Choroid plexus papilloma located in the trigone of lateral ventricle was found during the operation and was also removed (Fig. 2F). Postoperative reexamination of brain CT showed that most of the intracerebral hematoma was cleared, and the aneurysm clip was in good position (Fig. 1D–F). On the second day after the operation, the patient's consciousness gradually improved. On the 8th day after the operation, the reexamination of cerebral CT showed that the right intracerebral hemorrhage was cleared, aneurysm clip was visible, the left ventricle and subarachnoid hemorrhage were visible (Fig. 1G–K), and the size of bone window was about 3cm (Fig. 2L). To discharge intraventricular and subarachnoid hematoma and decomposition products as soon as possible, reduce the incidence of complications such as fever and communicating hydrocephalus, lumbar cistern drainage was performed, and gentamicin and dexamethasone were injected into the sheath. After 6 days of continued lumbar cistern drainage, the fever of the patient improved, and the bloody cerebrospinal fluid gradually changed into colorless and transparent cerebrospinal fluid. Then the lumbar cistern drainage tube was removed. Pathological examination showed aneurysm (Fig. 2M) and choroid plexus papilloma (Fig. 2N). Brain CT reexamination before discharge showed that cerebral hemorrhage was completely absorbed, and no obvious hydrocephalus was found (Fig. 1L–N). The patient was conscious at discharge and had no obvious neurological dysfunction. One month after hospital discharge, the telephone follow-up did not reveal any special abnormalities. No evidence of the symptom recurrence.

## 3 Discussion

Pure intravascular hemorrhage accounts for about 3.3% of all intracranial hemorrhage, of which about 70% is secondary. The main causes include hypertension, moyamoya disease, aneurysm, arteriovenous malformation, etc., which often leads to acute obstructive hydrocephalus and endangers life [6]. Generally, emergency surgery is required, and the relevant vascular examination is essential for a definite diagnosis. Recently, we reported a rare case of pure intraventricular hemorrhage secondary to rupture of a distal segment aneurysm of the anterior choroidal artery. This patient was also complicated with puff of smoke sign and choroidal papilloma. We used transcranial neuroendoscopy for minimally invasive craniotomy, including one-time clipping of the aneurysm, removal of intraventricular hematoma and removal of choroidal papilloma. The patient's preoperative cerebral CT examination found pure intraventricular hemorrhage, and the emergency treatment was planned to be performed by transcranial neuroendoscope assisted intraventricular hematoma removal through the frontal approach of the lateral ventricle. Fortunately, we completed the head and neck CTA examination in the emergency treatment and found that the patient had a distal segment aneurysm of the right anterior choroidal artery, so the surgical approach and method were changed in time. According to the location of the aneurysm reconstructed by 3D slicer, we chose the lateral ventricular triangle approach to clamp the aneurysm and remove the intraventricular hematoma.

Ali et al. [4]showed that the mortality rate of patients with moyamoya disease with first bleeding was about 5%, but the mortality rate of rebleeding was as high as 25%. In patients with the same site of rebleeding, the main cause of bleeding is aneurysmal rupture. In patients with different sites of rebleeding, the main cause of bleeding is the rupture of malformed vessels under abnormal blood flow pressure. Therefore, in the treatment of patients with intraventricular hemorrhage complicated by moyamoya disease and aneurysms, the risk of early rebleeding and high mortality should be considered, and active surgical treatment should be taken early to remove the risk of rebleeding. For patients with aneurysms at the distal segment of such arteries, it is extremely difficult to accurately locate them. It is not suggested to search for the aneurysm from the origin of the parent artery to the far end, because it will cause greater secondary injury. Our team reconstructed the aneurysm, ventricular system, and intracranial structures through 3D Slicer to accurately display the location of the aneurysm in the ventricle and the projection of the scalp on the body surface for positioning, which is very easy and accurate to locate the aneurysm. By this means, we can minimize the size of skin incision, optimize craniotomy, and theoretically have the least impact on normal brain tissue and collateral circulation. If the parent artery is an important vessel and it is difficult to retain the parent artery in preoperative evaluation, preoperative bypass surgery should be considered [4].

Some other scholars suggest conservative treatment. They believe that these aneurysms are pseudoaneurysms and tend to disappear spontaneously during follow-up [4]. Because the choroidal artery is a peripheral artery, lacks collateral circulation, and supplies key brain structures such as the basal ganglia and optic tract, surgical treatment of distal choroidal artery aneurysms is extremely challenging and dangerous. At present, there are three surgical methods for such aneurysms, including routine microsurgery, intravascular interventional therapy and neuroendoscopic surgery [3]. Although conventional microsurgery is technically mature, it has disadvantages such as large trauma and serious complications, which is not the best choice for aneurysms at the distal segment of the artery that are not easy to locate accurately. And the average diameter of choroidal artery is about 0.94mm (0.7–1.2mm) [7], which is in the deep part of the brain and has a tortuous path. So, the path of intravascular interventional therapy is long and difficult to reach, which is a great test for the operator and surgical materials. It is difficult to successfully operate without causing secondary injuries. Transcranial neuroendoscope minimally invasive surgery technology, using the advantages of neuroendoscope such as good light, flexible freedom, and close observation, has natural advantages in intracerebral hemorrhage surgery [[8], [9], [10]]. It is undoubtedly the best choice to cooperate with our team's newly developed 3D slicer reconstruction and precise positioning system [11,12]. In this case, we preliminarily decided to choose the frontal horn approach of lateral ventricle to clear the intraventricular hematoma according to the cerebral CT results of the patient in the external hospital. If we had done that, we would not only have been unable to deal with the posterior lateral choroidal aneurysm, but also have caused massive hemorrhage during the operation, which would have endangered the safety of the patient. Therefore, we urgently carried out the cerebral CTA before operation to know the existence of aneurysms, and located the relationship between aneurysms, ventricular system, and scalp location through 3D slicer reconstruction. Through rational preoperative design and planning, the surgical approach was changed in time, the operation was completed safely, and good results were achieved. The patient was conscious and had no obvious neurological dysfunction when discharged from the hospital.

## 4 Conclusion

Pure intraventricular hemorrhage requires vigilance against moyamoya disease and aneurysms at the distal segment of choroidal artery. It is very important to complete cerebral CTA before operation for the safety of operation. Because of the importance of choroidal artery, the surgical treatment of distal choroidal artery aneurysms is extremely challenging and dangerous. At present, conventional microsurgery and intravascular interventional therapy have limitations. Transcranial neuroendoscopic minimally invasive surgery has the advantages of good light, flexible freedom, and close observation. Combined with 3D slicer reconstruction and precise positioning system [11,12], it may be a good choice for the treatment of distal terminal artery aneurysms that lack collateral circulation.

## Production notes

### Author contribution statement

All authors listed have significantly contributed to the investigation, development and writing of this article.

### Data availability statement

Data will be made available on request.

### Funding

This work was supported by National Natural Science Foundation of China (81671306; 81971158; 82271518), Wuhan Science and Technology project (2019020701011470).

### Ethics approval statement

All procedures performed in studies involving human participants were in accordance with the ethical standards of the institutional and/or national research committee and with the 1964 Helsinki declaration and its later amendments or comparable ethical standards. This study was approved by the ethics committee of Clinical Research, Renmin Hospital of Wuhan University (WDRY2022-KS003), and the patient and his family members signed informed consent.

### Patient and public involvement’ statement

No patient involved.

## Declaration of competing interest

The authors declare that they have no known competing financial interests or personal relationships that could have appeared to influence the work reported in this paper.
